# Signature of topological states in antiferromagnetic Sm-substituted Bi_2_Te_3_

**DOI:** 10.1038/s41598-020-66521-9

**Published:** 2020-06-15

**Authors:** Jin-Hyeon Jun, Jinsu Kim, Soo-Whan Kim, Myung-Hwa Jung

**Affiliations:** Department of Physics, Sogang University, Seoul, 04107 South Korea

**Keywords:** Topological insulators, Topological insulators

## Abstract

An antiferromagnetic topological insulator has been predicted to be preserved by breaking both time-reversal symmetry and primitive lattice translational symmetry. However, the topological surface state has often been observed to disappear in an antiferromagnetic phase because the doped magnetic impurity acts as an extrinsic defect. In this study, we report the experimental signature of topological surface states coexisting with antiferromagnetic order in Sm-doped Bi_2_Te_3_. We fabricate single crystals of Sm_*x*_Bi_2−*x*_Te_3_ with *x* = 0.004, 0.010, and 0.025, where the Curie-Weiss law is satisfied at low temperatures but is violated at high temperatures due to the influence of the high energy states of *J* multiplets of Sm. For *x* = 0.025, e xotic physical properties are observed, such as the antiferromagnetic phase with the Néel temperature *T*_N_ = 3.3 K, multi-band Hall effect with two conduction channel, and anisotropic Shubnikov-de Haas oscillations. In the antiferromagnetic phase, we detect the signature of nontrivial topological surface states with surface electron density *n*_s_ = 7.9 × 10^11^ cm^−2^ and its high mobility *μ*_s_ = 2,200 cm^2^/Vs, compared to *n*_b_ = 2.0 × 10^19^ cm^−3^ and *μ*_b_ = 2.3 cm^2^/Vs for bulk electrons. These observations suggest that Sm_*x*_Bi_2−*x*_Te_3_ is a candidate creating the new stage for the potential application of topological antiferromagnetic spintronics.

## Introduction

Topological insulators (TIs) have attracted attention due to the peculiar band structure of insulating bulk bands coexisting with conducting surface states^1–3^. The time-reversal symmetry allows Dirac quasiparticles of surface states to have spin-momentum locking and to be protected against backscattering from non-magnetic scatters^4,5^. For realizing the quantum anomalous Hall effect in TIs^6–8^ and for providing spintronic applications to use the topologically enhanced spin-orbit torque^9–11^, there have been many studies on the introduction of ferromagnetism into topologically nontrivial states. Ferromagnetic TIs have been proposed by doping magnetic impurities such as transition metals^7,8,12^ or by proximity coupling at the interface of ferromagnet/TI bilayer systems^13,14^. The transition metal, which acts as a ferromagnetic source, in general breaks the time-reversal symmetry and leads to gap opening^15^. The gapped surface states are important to realize many exotic phenomena such as the quantum anomalous Hall effect and the axion electrodynamics^6–8,15,16^. However, the ferromagnetic source of doped transition metals such as Fe and Mn can make the topologically nontrivial states unstable in an extreme case by creating hole dopants into the system, because the transition metals are divalent. Alternatively, antiferromagnetic TIs have begun to be of interest as another class of topological matters, because the antiferromagnetism introduced by magnetic dopants to TIs cannot disturb the topologically nontrivial states and it has an advantage to reduce the critical current density in spintronic devices. A recent study has proposed that antiferromagnetic resonance can be electrically driven due to its topological magnetoelectric effect in antiferromagnetic TIs^17^.

For an issue as to how the antiferromagnetic order interplays with the topologically nontrivial states, there are two different theoretical ways. One is about the intrinsic antiferromagnetic TIs, where the topological surface state and antiferromagnetism can coexist by breaking both time-reversal symmetry and primitive lattice translational symmetry but preserving the product of those symmetries^18–20^. The other reports how correlations affect the topological surface states, where the topological insulating phase becomes a topologically trivial antiferromagnetic phase due to large Coulomb repulsion^21^. Those theoretical conclusions have been still poorly verified experimentally. Our previous works have shown that antiferromagnetic order in Ce- and Gd-doped Bi_2_Se_3_ and Bi_2_Te_3_ samples are strongly competing with topological surface states^22–24^. Here it is noteworthy that the rare-earth elements of Ce and Gd are expected to tune only the magnetism, not affect the electrical transport because they are mostly isovalent to the Bi atom and comparable in size. In theoretical aspect, some candidates such as GdBiPt and pressurized SmB_6_ have been proposed as antiferromagnetic TIs^18,25,26^. Very recently, MnBi_2_Te_4_ has been highly explored both theoretically and experimentally as an antiferromagnetic TI^27–31^, in which the linear energy dispersion of topological surface states with a small gap is clearly observed in the antiferromagnetic phase.

In this study, we report the signature of topological states in antiferromagnetic Sm-substituted Bi_2_Te_3_, focusing on how the topological surface states evolve with the magnetic dopant of Sm. The Sm element is different from other rare-earth elements due to the influence of the high energy states of *J* multiplets because the interval between the multiplets is small enough to be thermally excited^32^, which causes the violation of the Curie-Weiss law at high temperatures. As the Sm concentration *x* increases in Sm_*x*_Bi_2−*x*_Te_3_ (*x* = 0.004, 0.010, and 0.025), we find a transition to the antiferromagnetic phase with the Néel temperature *T*_N_ = 3.3 K for *x* = 0.025, where we observe large and linear magnetoresistance, multi-band Hall effect, and anisotropic Shubnikov-de Haas (SdH) oscillations. From the analyses of SdH oscillations, we detect the signature of nontrivial topological surface states pointing to π Berry phase shift in the antiferromagnetic phase. These observations suggest that the antiferromagnetic order can coexist with the topologically nontrivial states in Sm-doped Bi_2_Te_3_, contrary to other rare-earth doped TIs.

## Results and discussion

**Figure 1:**
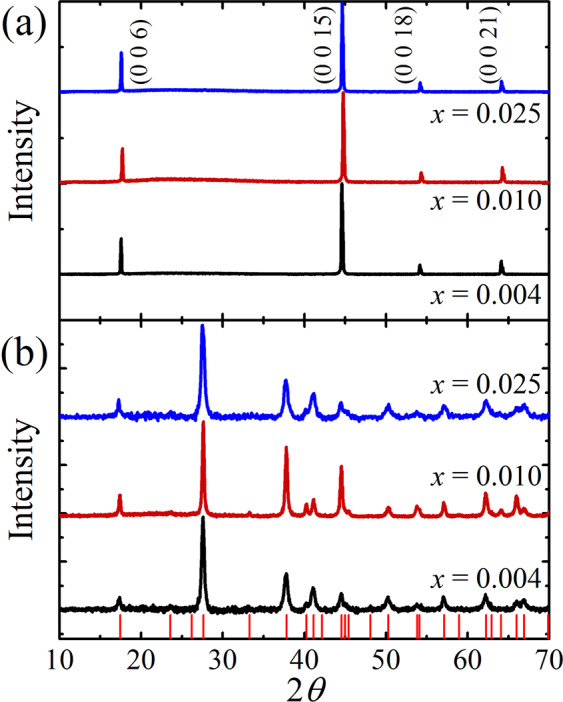
(**a**) Single-crystal X-ray diffraction patterns and (**b**) powder X-ray diffraction patterns for Sm_*x*_Bi_2−*x*_Te_3_ (*x* = 0.004, 0.010, and 0.025). The red solid lines represent the reference peaks of Bi_2_Te_3_.

Single crystals of Sm_*x*_Bi_2−*x*_Te_3_ are characterized by single-crystal and powder X-ray diffraction (XRD) experiments. As shown in Fig. 1a, the single-crystal XRD peaks for all the samples are well labeled with (00 L) indices of the plane perpendicular to the *c*-axis of the rhombohedral Bi_2_Te_3_ crystal structure. These results indicate that the inserted Sm does not disturb the crystal structure of pristine Bi_2_Te_3_ and that all the samples are sufficiently *c*-axis oriented single crystals. The *c*-axis lattice parameter evaluated from the peak positions is *c* = 30.44 Å, which is identical to the value reported previously in pristine Bi_2_Te_3_^33^. We also plot the powder XRD patterns in Fig. 1b, where no secondary or impurity peaks are found. There is no peak shift observed with varying the Sm contents *x*, indicating no variations on lattice parameters, and the obtained lattice parameter of *a* = 4.39 Å is also consistent with other literature^33^. These observations suggest that Sm is well substituted for the Bi site without any lattice distortion in a single crystal form.

**Figure 2:**
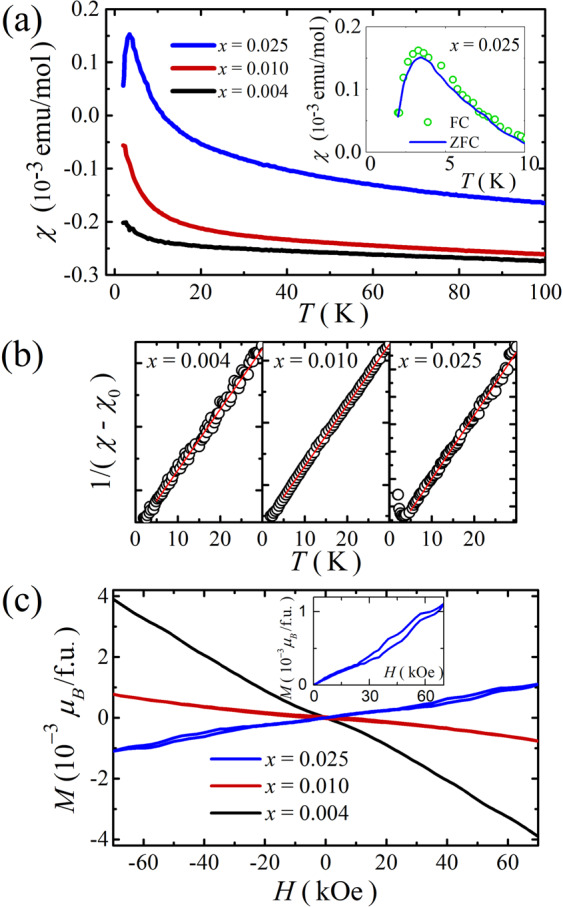
(**a**) Temperature dependence of magnetic susceptibility *χ*(*T*) for Sm_*x*_Bi_2−*x*_Te_3_ (*x* = 0.004, 0.010, and 0.025) measured in a field of 15 kOe after zero-field cooling. The inset shows zero-field cooled and field cooled curves for *x* = 0.025 in a field of 15 kOe. (**b**) The best fits of the modified Curie-Weiss law at low temperatures below 30 K. (**c**) Field dependence of magnetization *M*(*H*) curves for Sm_*x*_Bi_2−*x*_Te_3_ (*x* = 0.025, 0.010, and 0.004) measured at 2 K. The inset shows magnified curve at *x* = 0.025 clarifying the field-induced metamagnetic transition.

In order to investigate the substituted Sm amount in Sm_*x*_Bi_2−*x*_Te_3_, we measure the temperature dependence of magnetic susceptibility *χ*(*T*). Figure 2a shows the *χ*(*T*) curves for Sm_*x*_Bi_2−*x*_Te_3_. The magnetic moment gradually increases with increasing *x*, although the diamagnetic signal of the order of 10^−4^ emu/mol is dominant for all the samples. Since Bi_2_Te_3_ gives a predominant diamagnetic contribution of magnetic susceptibility, the gradual increment in the magnetic moment is likely to come from the paramagnetic contribution by Sm substitution. Thus, the *χ*(*T*) data minus the temperature-independent term *χ*_0_ should follow the Curie-Weiss law; *χ*(*T*)−*χ*_0_ = *C*/(*T*−*θ*_P_)^34^, where *C* is the Curie constant and *θ*_P_ is the Weiss temperature. Here it is noteworthy that the Curie-Weiss law is not satisfied in the high temperature regime owing to the population of excited *J* multiplet states of Sm. Instead, the *χ*(*T*) data are well fitted at low temperatures below 30 K, and the best fitted curves are plotted in Fig. 2b. From the linear fit of 1/(*χ*−*χ*_0_) vs. *T*, we can obtain *χ*_0_, *θ*_P_ from the intercept, and *C* from the slope. The Curie constant is related to the effective magnetic moment, *C* = *xμ*_eff_^2^/3*k*_B_, where *k*_B_ is the Boltzmann constant, *μ*_eff_ is the effective magnetic moment, and *x* is the number of magnetic atoms per formula unit. Using the theoretical value of effective magnetic moment (0.85 *μ*_B_) for free Sm^3+^ ion^34^, we obtain *x* = 0.004, 0.010, and 0.025. Based on these results, the *x* values are described in the text. The best fits yield *χ*_0_ = −2.61 × 10^−4^, −2.54 × 10^−4^, and −1.43 × 10^−4^ emu/mol, *θ*_P_ = −2.4, −2.5, and −3.3 K, and *C* = 0.4 × 10^−3^, 1.0 × 10^−3^, and 2.2 × 10^−3^ emu K/(mol Oe) for *x* = 0.004, 0.010, and 0.025, respectively. Here, the ionic state of Sm substituted for Bi^3+^ is presumably to be Sm^3+^. If Sm^2+^ is substituted to Bi^3+^, the lattice parameter increases, and the XRD peak position shifts to a lower angle, because the ionic radius of Sm^2+^ (122 pm) is larger than that of Sm^3+^ (102 pm), compared to Bi^3+^ (103 pm)^35^. However, the XRD data show no peak shift, indicating no change of lattice parameters. In addition, if Sm^2+^ is substituted for Bi^3+^, there is an additional transport effect to generate hole dopants. However, the carrier density calculated from the Hall measurements is almost same. Therefore, the most possible ionic state of Sm seems to be Sm^3+^ in this compound. For *x* = 0.025, the most noticeable feature in *χ*(*T*) is a sharp peak at *T*_N_ = 3.3 K. This peak structure is a feature of antiferromagnetic transition, rather than spin glass behavior, because the zero-field cooled and field-cooled curves are the same below *T*_N_, as seen in the inset of Fig. 2. The magnetic state for *x* < 0.025 is not clear at least for the measured temperature range between 2 and 300 K. Even if there is an antiferromagnetic transition in *x* = 0.004 and 0.010, we could not observe the transition in the measured temperature range.

For further study on the magnetic states with varying *x*, we also measure the field dependence of magnetization *M*(*H*) at 2 K. As seen in Fig. 2c, the magnetization increases from the negative to positive slope with increasing *x*, in good agreement with the *χ*(*T*) results. As expected, for *x* = 0.025, a metamagnetic transition from the antiferromagnetic to spin-flop phase occurs in the vicinity of 3 T with hysteresis. This result is compared to the previous report on Sm-doped Bi_2_Se_3_, where a ferromagnetic transition is observed^36^.

**Figure 3:**
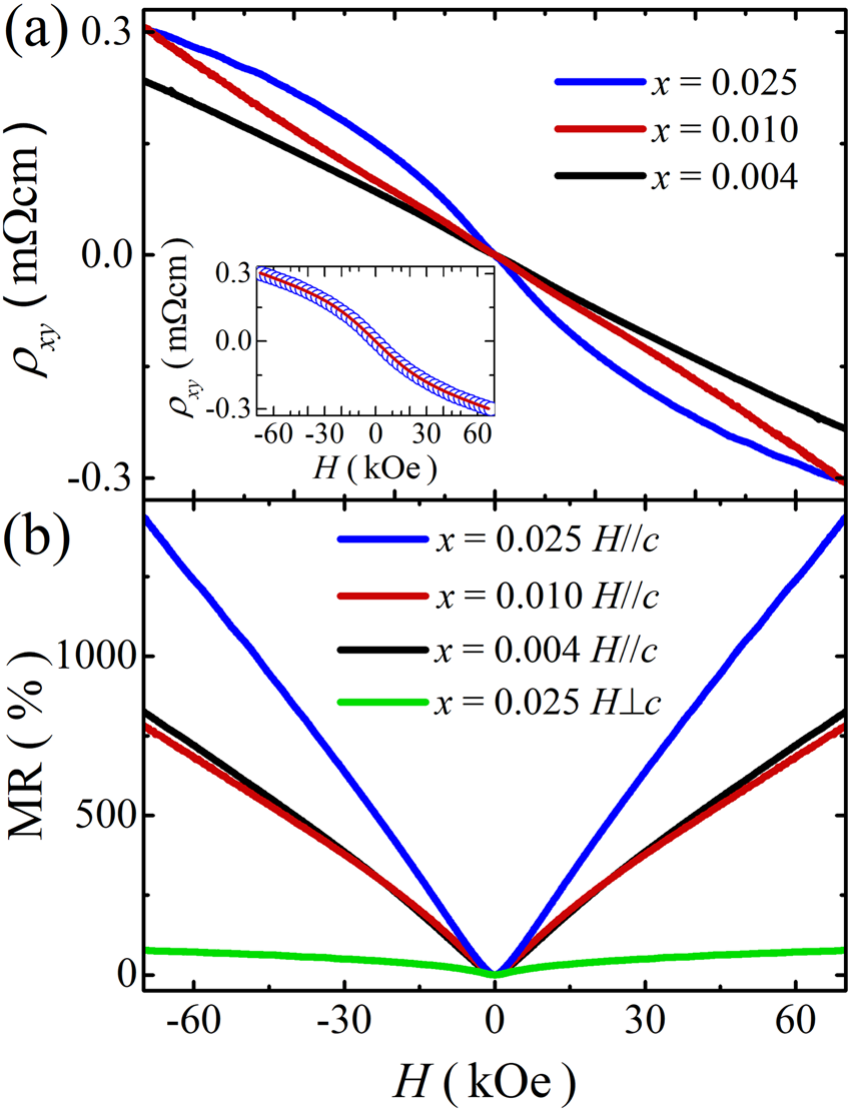
(**a**) Hall resistivity *ρ*_*xy*_(*H*) at 2 K for Sm_*x*_Bi_2−*x*_Te_3_ (*x* = 0.004, 0.010, and 0.025) with a magnetic field applied along the *c*-axis. The inset shows the comparison between the experimental data at *x* = 0.025 and the fitted curve with two-band model. (**b**) Magnetoresistance ratio, MR = 100 × [*ρ*_*xx*_(*H*) − *ρ*_*xx*_(0)]/*ρ*_*xx*_(0) at 2 K for Sm_*x*_Bi_2−*x*_Te_3_ (*x* = 0.004, 0.010, and 0.025) with a magnetic field parallel and perpendicular to the *c*-axis (*H*//*c* and *H*⊥*c*).

In the topological insulating system, the magnetic phase may cause a significant change in electronic properties due to the correlation between magnetism and the topological surface states. Figure 3a shows the Hall resistivity *ρ*_*xy*_(*H*) at 2 K as a function of the magnetic field. All the samples display negative slopes that originate from n-type carriers. For *x* = 0.004 and 0.010, the *ρ*_*xy*_(*H*) curves are linear, from which the carrier density is estimated to be 1.8 × 10^19^ and 1.5 × 10^19^ cm^−3^ for *x* = 0.004 and 0.010, respectively. However, for *x* = 0.025, the *ρ*_*xy*_(*H*) curve is not a linear function, which can be interpreted by the multi-band Hall effect expected in a two-band topological insulating system that possesses both bulk and surface carriers in parallel^4,37,38^. The Hall resistivity can be expressed as the standard two-band model^4,37^,

$${\rho }_{xy}=\frac{({R}_{s}{\rho }_{b}^{2}+{R}_{b}{\rho }_{s}^{2}){\mu }_{0}H+{R}_{s}{R}_{b}({R}_{s}+{R}_{b}){({\mu }_{0}H)}^{3}}{{({\rho }_{s}+{\rho }_{b})}^{2}+{({R}_{s}+{R}_{b})}^{2}{({\mu }_{0}H)}^{2}}$$

where ${\mu }_{0}$ is the magnetic permeability of vacuum, ${\rho }_{b}$ and ${R}_{b}$ are the electrical resistivity and Hall coefficient of the bulk electrons, and ${\rho }_{s}$ (=*R*_*□*_ *t*) and (*R*_*S*_=*t*/e*n*_s_) are the electrical resistivity and Hall coefficient of the surface carriers with *R*_□_ the surface sheet resistance and *t* the sample thickness. The best fit of the two-band analysis of the Hall curve in the inset of Fig. 3a reveals that the surface carrier density is deduced to be *n*_s_ = 7.9 × 10^11^ cm^−2^ and its mobility is *μ*_s_ = 2,200 cm^2^/Vs, compared to the bulk carrier density of *n*_b_ = 2.0 × 10^19^ cm^−3^ and its mobility of *μ*_b_ = 2.3 cm^2^/Vs. These fitting parameters are consistent with the previous result in TIs with two conduction channels^37^. The results suggest that surface-band transport is dominant in the antiferromagnetic material of *x* = 0.025. Although all the samples of Sm_*x*_Bi_2−*x*_Te_3_ (*x* = 0.004, 0.010, 0.025) have a similar bulk carrier density ~10^19^ cm^−3^, it is noticeable that the topological surface properties are prominent only at *x* = 0.025.

We further investigate the field-dependent resistivity *ρ*_*xx*_(*H*), which gives more information on the topological surface properties. Figure 3b shows the magnetoresistance ratio, MR = 100 × [*ρ*_*xx*_(*H*) − *ρ*_*xx*_(0)]/*ρ*_*xx*_(0) at 2 K for all the samples of Sm_*x*_Bi_2−*x*_Te_3_. All the MR curves increase linearly, and the linear MR behavior is most likely to be related to the surface electrons with linear energy dispersion^39–41^. Since there is no magnetic order coexisting with the topological surface states in *x* = 0.004 and 0.010, the MR curves are linear. Notably, the MR curve for *x* = 0.025 increases more than the other two samples. This enhanced linear MR and the nonlinear Hall curve for *x* = 0.025 are important features where the topological surface states are around the Fermi level^39^. Therefore, we suggest that for *x* = 0.025 with the antiferromagnetic order, the topological surface states still survive. Further experiments at lower temperatures below 2 K are required to investigate possible magnetic and topological states for lower Sm contents (*x* = 0.004 and 0.010).

**Figure 4:**
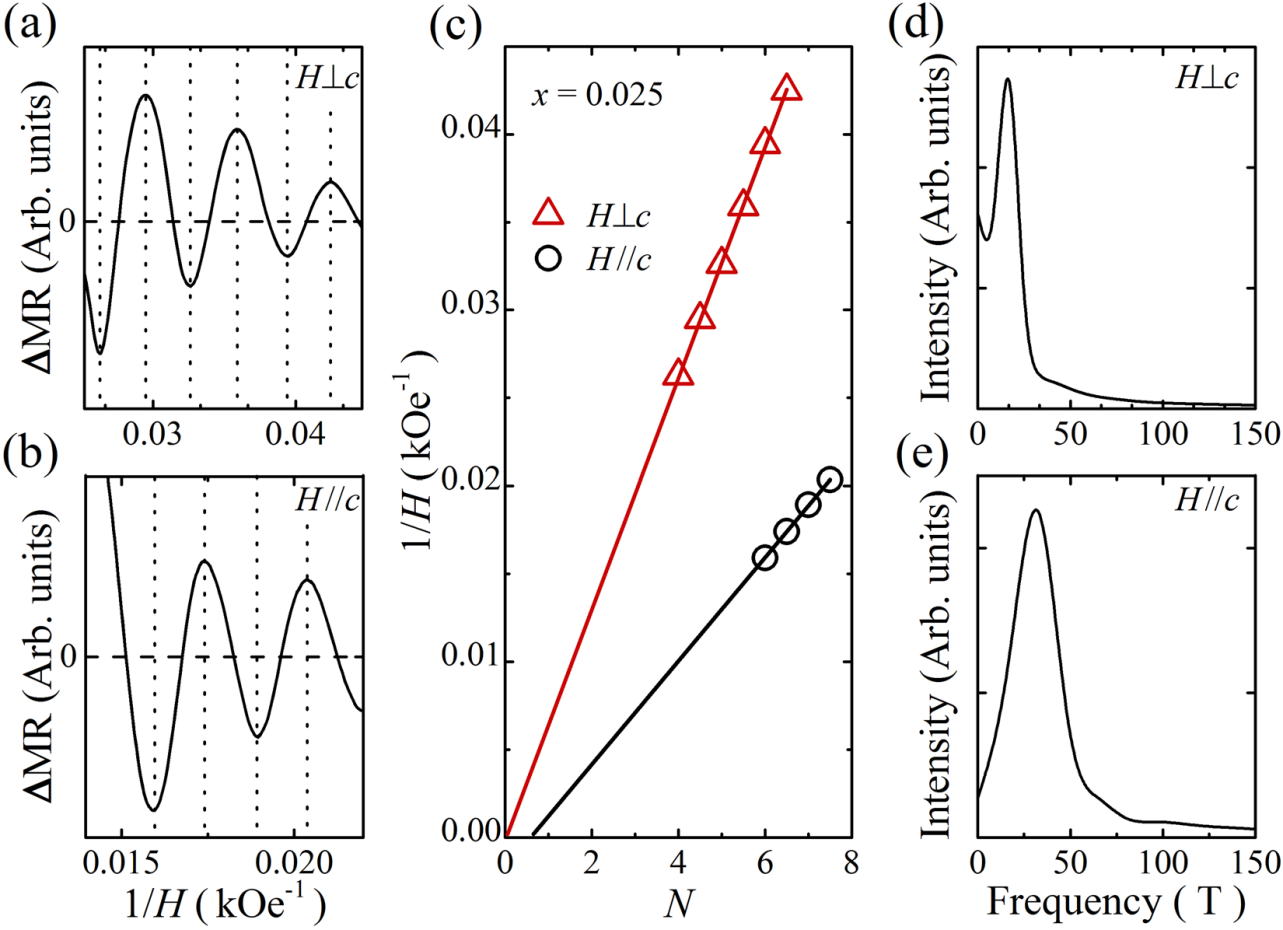
Subtracted SdH oscillations at 2 K measured in (**a**) *H*⊥*c* and (**b**) *H*//*c* configurations, respectively. (**c**) Landau level fan diagram obtained from the SdH oscillations for both *H*⊥*c* and *H*//*c*. (**d**,**e**) Fast Fourier transform results of the SdH oscillations presented in panels (a,b), respectively.

A careful look at high-field data gives SdH oscillations due to the formation of quantized Landau levels in high magnetic fields. On subtracting a polynomial fit to the background signal, we obtain clear oscillating signals for the magnetic field perpendicular and parallel to the *c*-axis (between 45 and 70 kOe for *H*//*c* and between 20 and 45 kOe for *H*⊥*c*) in Fig. 4a,b. Note that the oscillating signals in *x* = 0.004 and 0.010 are not resolved. In order to carefully analyze the SdH oscillations, we convert the MR data to the conductivity σ_*xx*_ = *ρ*_*xx*_/(*ρ*_*xx*_^2^ + *ρ*_*xy*_^2^), as done in previous studies^4,37^, and extract the Landau level fan diagram shown in Fig. 4c. We find two oscillation frequencies, which provide different Berry phases. The straight-line extrapolation gives a Berry phase factor *β* = 0.02 and 0.64 for *H*⊥*c* and *H*//*c*, respectively, that is the intersection point on the Landau-level index *N* axis. The anisotropy of the Berry phase supports the existence of topologically nontrivial states due to the 2-dimensional Fermi surface nature^4^. For *H*⊥*c*, *β* is close to 0, which is the case when the Berry phase vanishes in parabolic energy dispersion. The *β* value (=0.64) obtained from the SdH analysis for *H*//*c* is close to the value of *β* = 0.5 for the ideal massless Dirac fermions with the nature of 2-dimensional Fermi surface in origin, implying that the quantum oscillation for *H*//*c* is due to the topological surface electrons with linear energy dispersion. The deviation from the ideal *β* value has been understood by the hybridization and/or mixing of the linear surface band with the quadratic bulk band^42^. The oscillation frequencies are *F* = 15.3 T and 32.0 T for *H*⊥*c* and *H*//*c*, respectively, which coincide with the fast Fourier transform results seen in Fig. 4d,e, implying two different Fermi surfaces contributing to the electrical conduction. It is plausible that one for *H*⊥*c* corresponds to the bulk dominant, and the other for *H*//*c* is surface dominant. Considering a simple circular Fermi surface, from the oscillation frequency for *H*//*c* we estimate the surface carrier density *n*_s_ = 7.9 × 10^11^ cm^−2^, which is the same as the value used in the two-band Hall analysis. Considering a cylindrical Fermi surface for *H*⊥c, we calculate the bulk carrier density *n*_b_ = 2.0 × 10^19^ cm^−3^, in the same order of magnitude as obtained from the two-channel analysis of Hall data.

In conclusion, we prepared single crystals of Sm_*x*_Bi_2−*x*_Te_3_, in which the actual composition of *x* (= 0.004, 0.010 and 0.025) is determined by the Curie-Weiss law of the magnetic susceptibility data at low temperatures. The XRD results indicated that the inserted Sm does not disturb the crystal structure of pristine Bi_2_Te_3_ and that all the samples are sufficiently *c*-axis oriented single crystals. The magnetization data revealed that an antiferromagnetic transition occurs at *T*_N_ = 3.3 K only for *x* = 0.025, where the transport data exhibit not only two-band conduction channels of both surface and bulk electrons but also anisotropic SdH oscillations for the magnetic field directions parallel and perpendicular to the *c*-axis. The important finding is that the antiferromagnetic order can coexist with the topologically nontrivial states in Sm_*x*_Bi_2−*x*_Te_3_, contrary to other magnetic TIs, which suggests a promising potential for topological antiferromagnetic spintronics.

## Methods

### Sample growth and characterization

The single crystals of Sm-substituted Bi_2_Te_3_ (Sm_*x*_Bi_2−*x*_Te_3_) were grown by melting method with mixtures of high-purity Sm (99.9%), Bi (99.999%), and Te (99.999%). The mixtures sealed in evacuated quartz tubes were heated to 920 °C for 1 day and kept for 5 days at this temperature. After heating, they were slowly cooled down to 520 °C over 15 days, annealed for 5 days, and then finally cooled down to room temperature. All the obtained crystals were well cleaved with a silvery, flat, and mirror-like surface perpendicular to the *c*-axis. The crystal structure was confirmed by an X-ray diffractometer with Cu Kα radiation.

### Magnetic and electrical property measurements

The electrical transport and magnetic properties were investigated with a superconducting quantum interference device-vibrating sample magnetometer (SQUID-VSM) up to 70 kOe in the temperature range down to 2 K. The magnetic data were taken in an applied magnetic field of 15 kOe after cooled with zero field (called zero-field cooled process) and after cooled with the magnetic field (called field cooled process). Both curves in Fig. 2 are measured in a field of 15 kOe. The only difference is the cooling process; one is cooled in zero field, and the other is cooled in a field. All electrical and magnetic measurements were carried out in the magnetic field perpendicular or parallel to the cleaved surface of samples. The thickness of our samples used in transport measurements is 0.26, 0.51, and 0.28 mm for *x* = 0.004, 0.01, and 0.025, respectively.

## Data Availability

The datasets analyzed during the current study are available from the corresponding author on reasonable request.
